# Case Report: A case of postneurosurgery *Klebsiella pneumoniae* meningitis with longitudinal CSF cytokine profiling and combined intrathecal therapy

**DOI:** 10.3389/fimmu.2026.1945662

**Published:** 2026-10-15

**Authors:** Lin Zhu, Qian Wu, Jing Xu, Deshan Ge, Shujuan Dai

**Affiliations:** 1 Department of Rehabilitation, The Third People’s Hospital of Yunnan Province, Kunming, Yunnan, China; 2 Department of Neurology, First Affiliated Hospital, Kunming Medical University, Kunming, Yunnan, China

**Keywords:** cytokines, interleukin-1β, intrathecal therapy, *Klebsiella pneumoniae*, meningitis

## Abstract

A 47-year-old woman with poorly controlled diabetes developed acute fever, headache, seizures, and progressive confusion during an admission for glycemic management. Neurological examination showed mild meningeal signs, and brain MRI demonstrated diffuse linear leptomeningeal enhancement. Lumbar puncture revealed strikingly viscous, ‘jelly-like’ cerebrospinal fluid (CSF), and culture identified *Klebsiella pneumoniae*. Despite systemic antimicrobial therapy, central inflammatory activity persisted. Serial CSF analyses revealed pronounced elevations of IL-1β, IL-6, and IL-8, with IL-1β closely paralleling CSF leukocyte dynamics and demonstrating relative independence from systemic inflammatory fluctuations. Clinical and laboratory improvement occurred after combined intravenous and intrathecal administration of polymyxin B, amikacin, and dexamethasone, leading to full neurological recovery. This case highlights the diagnostic significance of mucoid CSF in *Klebsiella pneumoniae* meningitis and underscores the therapeutic value of intrathecal antibiotics in severe central nervous system infections. Longitudinal CSF IL-1β measurements may provide a potentially useful marker of intracranial inflammatory activity and treatment response, although this requires validation in larger cohorts.

## Introduction

*Klebsiella pneumoniae (also known as K. pneumoniae)* is a gram-negative bacterium belonging to the Enterobacteriaceae family. There are two major types of *K. pneumoniae*: classical *K. pneumoniae* and hypervirulent *K. pneumoniae*. Classical *K. pneumoniae* mainly affects hospitalized or immunocompromised patients with underlying diseases such as diabetes, malignancy, or recent surgery (1). Hypervirulent *K. pneumoniae* causes severe community-acquired infections in healthy individuals without known risk factors. The hypermucoviscous capsules, siderophore overproduction, and resistance to phagocytosis of hypervirulent *K. pneumoniae* enable it to more effectively breach the host barrier and evade immune responses compared to classical *K. pneumoniae* strains (2, 3). This leads to more widespread systemic diseases, including pneumonia, liver abscesses, meningitis, and necrotizing fasciitis. As an opportunistic pathogen, *K. pneumoniae* commonly colonizes mucosal surfaces such as the skin, nasopharynx, and gastrointestinal tract. Clinical infection typically occurs in immunocompromised individuals, those with chronic comorbidities, or following disruption of anatomical barriers via medical intervention (e.g., central venous catheters, endotracheal intubation, or neurosurgery) (4, 5).

*K. pneumoniae* meningitis is a life-threatening central nervous system (CNS) infection caused by the *K. pneumoniae*. Globally, *K. pneumoniae* meningitis is estimated to be the third leading cause of meningitis-related mortality (6), with reported case mortality rates ranging from 23.7% to 62.5% (7, 8). Given these high mortality rates, achieving effective antimicrobial concentrations within the CNS is paramount. Effective treatment of *K. pneumoniae* meningitis is often hindered by the limited penetration of systemic antibiotics across the blood-brain barrier (BBB), frequently resulting in subtherapeutic concentrations at the site of infection. Intrathecal or intraventricular antibiotic administration has emerged as a critical adjunctive strategy, enabling adequate cerebrospinal fluid (CSF) drug levels and improved outcomes, particularly in cases of post-neurosurgical meningitis (9, 10).

Here, we report a rare case of severe *K. pneumoniae* meningitis in patient with a history of transsphenoidal pituitary surgery, successfully managed through a combination of intravenous and intrathecal antimicrobial therapy. We present detailed serial CSF analyses, neuroimaging findings, and a comprehensive treatment timeline, offering practical insights into the diagnosis and management of this challenging condition.

## Case presentation

A 47-year-old women with a 6-year history of hyperglycemia was admitted to the endocrinology department for glycemic control. On hospital day 7, she developed recurrent febrile episodes (maximum temperature 38.6°C) and persistent headache, without associated seizures or altered consciousness. Oral acetaminophen was administered symptomatically.

On hospital day 11, the patient became unresponsive and exhibited generalized rigidity followed by a clonic seizure lasting 3–5 minutes. Postictally, she exhibited marked pallor and progressive obtundation, without urinary or fecal incontinence. Arterial blood gas analysis revealed metabolic acidosis: pH 7.20, lactate 9.2 mmol/L (reference range: 0.5-2.2 mmol/L), and glucose 9.9 mmol/L. Brain MRI and CT demonstrated calvarial defect in the left frontal-temporal region (**Figure 1**; **Supplementary Figure S1**). There was no clear evidence of paranasal sinus involvement or demonstrable communication between the sinonasal tract and intracranial compartment. In addition, patchy areas of encephalomalacia with surrounding gliosis were present in the left frontal and temporal lobes, consistent with postoperative changes. FLAIR imaging revealed diffuse linear leptomeningeal enhancement involving the bilateral frontal, temporal, parietal, and occipital lobes, cerebellar hemispheres, and brainstem. CNS infection was suspected, and the patient was transferred to the Neurological Critical Care unit (NCU).

**Figure 1:**
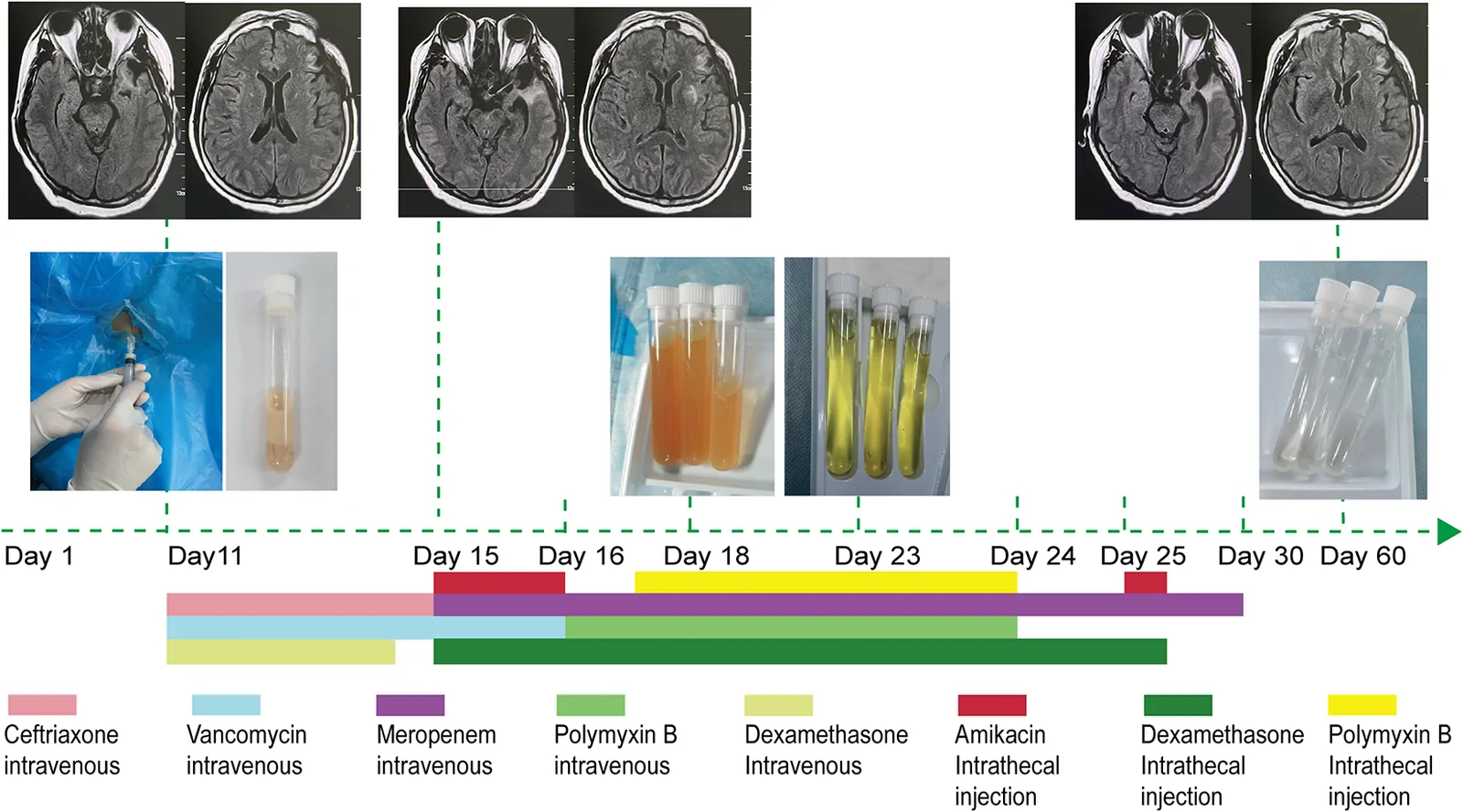
Clinical timeline, neuroimaging findings, and CSF appearance. Schematic overview of key clinical events, treatments, and neuroimaging results. Representative images showing the evolution of CSF appearance from turbid, viscous (‘jelly-like’) to clear.

### Past medical history

The patient had undergone transsphenoidal resection of a pituitary adenoma five years earlier at another institution, had no history of CSF leakage before this admission. One year prior to this admission, she had presented to the neurology department with persistent headache. At that time, brain MRI revealed similar diffuse leptomeningeal enhancement, and lumbar puncture showed elevated intracranial pressure (240 mmH_2_O) and significant leukocytosis (3438 x 10^6^/L). CNS infection was suspected, and she was treated empirically with antiviral agents (Acyclovir) and dehydration therapy. Her symptoms improved within two weeks, but she declined further evaluation and was discharged against medical advice.

### Neurological critical care unit admission (Day 11)

During transfer, the patient experienced another generalized tonic-clonic seizure lasting approximately 90 seconds, no incontinence was observed. On admission to NCU, the patient’s vital signs were: temperature 37.5 °C, heart rate 106 beats/min, blood pressure 114/79 mmHg, respiratory rate 24 breaths/min, and oxygen saturation 98%, Glasgow Coma Scale (GCS): E4V5M6. Neurological examination revealed: impaired calculation and memory, nuchal rigidity, and a positive Kernig’s sign. Laboratory studies showed leukocytosis (16.27×10^9^/L) with neutrophilia (11.84×10^9^/L), a normal lymphocyte count (3.94×10^9^/L), and elevated C-reactive protein (65.10 mg/L). Chest CT was unremarkable. Lumbar puncture yielded turbid, exceptionally viscous (‘jelly-like’) CSF; the opening pressure was unmeasurable due to the fluid’s consistency (**Figure 1**). All CSF samples obtained during hospitalization were collected through repeated lumbar punctures. Subsequent intrathecal medications were also administered via the lumbar intrathecal route during these procedures. No lumbar drain, ventricular access device, external ventricular drain, or other indwelling CNS hardware was present. A provisional diagnosis of CNS infection with secondary seizure activity was made. She was started on intravenous valproic acid, mannitol, ceftriaxone (day11–15: 2 g, q12h), vancomycin, dexamethasone (day11–14: 10 mg, q6h), and supportive care. Following microbiological confirmation of *K. pneumoniae* meningitis, serial measurements of inflammatory cytokines in both CSF and serum were performed to characterize the magnitude and persistence of the intracranial inflammatory response and to explore their potential utility for monitoring treatment response. These measurements were undertaken for longitudinal assessment rather than diagnostic confirmation.

### Subsequent course

On hospital day 15, the patient developed intermittent low-grade fever (36.3 °C - 38.5 °C). CSF culture (collected on day 11) grew *Klebsiella pneumoniae* (**Supplementary Table 1**), while concurrent sputum and blood cultures were negative. Temporal changes tested by multiplex flow cytometric immuno-bead array assay technology in CSF and serum leukocyte and cytokines (IL-1β, IL-2, IL-6, IL-8, IL-10, IL-17, TNF-α, IFN-γ) are shown in **Figure 2**. Serum inflammatory markers included CRP 20.80 mg/L (normal range: ≤6mg/L) and procalcitonin 1.01 ng/ml (normal range: < 0.05 ng/ml). Brain MRI findings remained largely unchanged, but with worsened diffuse leptomeningeal enhancement. Despite treatment with ceftriaxone (2 g, q12h), serial assessments demonstrated continued progression of central nervous system inflammation. CSF leukocyte counts increased, follow-up MRI showed worsening diffuse leptomeningeal enhancement, and subsequent CSF cultures remained positive for *K. pneumoniae*. Although the isolate was susceptible to ceftriaxone (**Supplementary Table 1**), the treating team considered these findings indicative of an inadequate clinical response and therefore escalated antimicrobial therapy to meropenem (day15–30: 2 g, q8h). Intrathecal dexamethasone (day15–26: 5 mg, q.o.d) and amikacin (day 15: 0.2 g; day 24: 0.05 g) were initiated.

**Figure 2:**
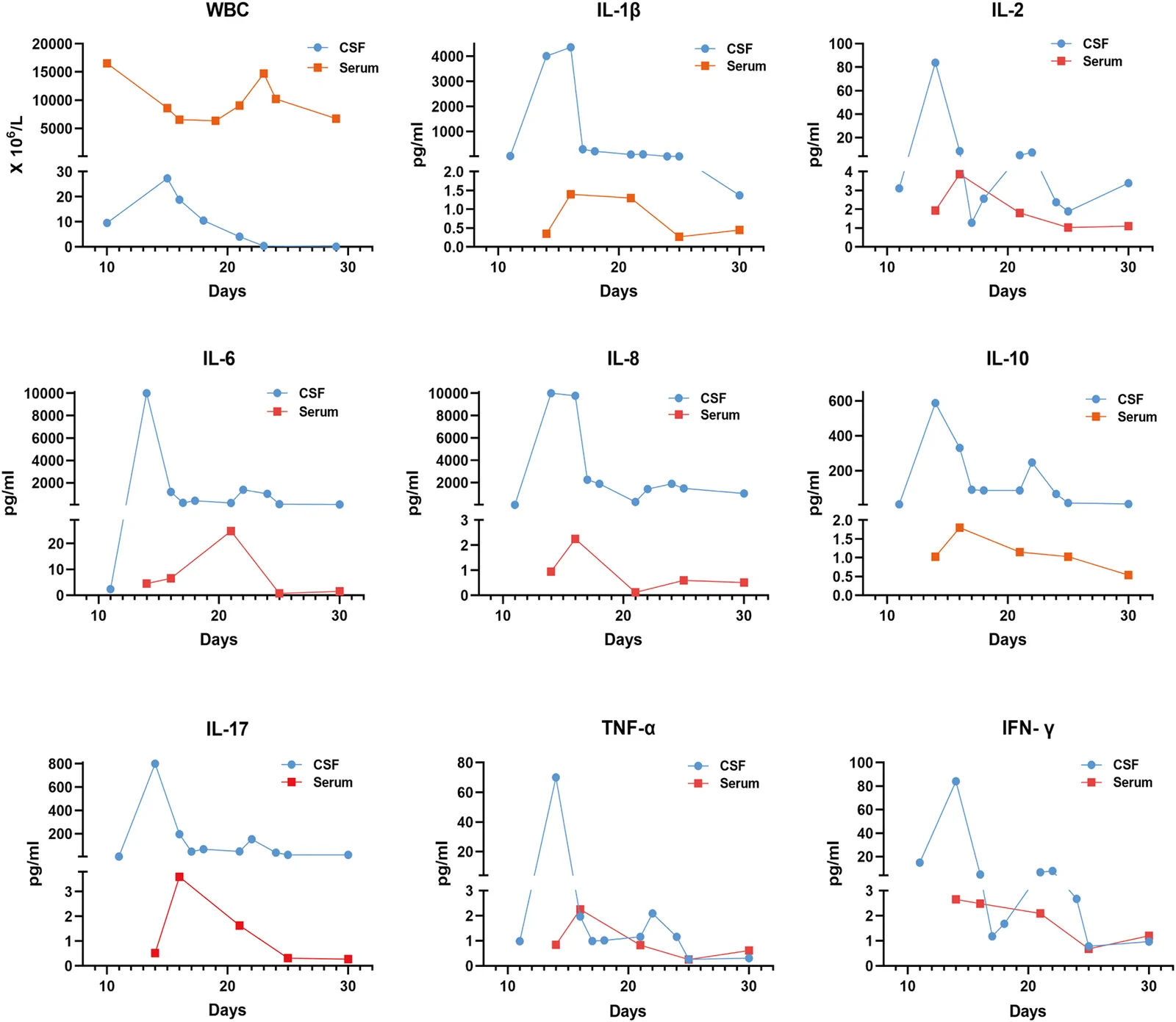
Longitudinal profiles of inflammatory markers. Temporal changes in leukocyte counts and selected cytokine (IL-1β, IL-2, IL-6, IL-8, IL-10, IL-17, TNF-α, IFN-γ ) concentrations in serum (red lines) and cerebrospinal fluid (CSF, blue lines).

By hospital day 16, her body temperature decreased (peak 37.9 °C). Given the ongoing inflammatory activity and microbiological confirmation of *K. pneumoniae*, intravenous polymyxin B (day 16–24) was added, followed by intrathecal polymyxin B administration (day 17: 10000 U; day 19: 30000 U; day 21-26, 50000 U, q.o.d). Serial CSF cultures were performed throughout hospitalization. *K. pneumoniae* was isolated from CSF specimens obtained on hospital days 11 and 17. All subsequent CSF cultures obtained after day 18 were negative.

### Outcome

By hospital day 30, the patient showed marked neurological improvement, including complete cessation of seizures and near-normal CSF parameters. Vital signs: temperature 36.3°C, HR 78 beats/min, RR 16 breaths/min, BP 112/67 mmHg, SpO2 98%; GCS: E4V5M6. Mild nuchal rigidity remained. Serial serum creatinine measurements remained stable throughout hospitalization, and no acute kidney injury or other evidence of renal dysfunction was observed (**Supplementary Figure S2**). She was discharged in stable condition.

At follow-up on day 60, the patient reported no discomfort or neurological symptoms. Neurological exams were normal. Brain MRI showed stable postoperative changes without abnormal enhancement. Lumbar puncture revealed clear CSF with normal opening pressure (150 mmH_2_O).

## Discussion

The patient had undergone transsphenoidal resection of a pituitary adenoma five years prior, without subsequent cranial reconstruction. One year before the current admission, she was hospitalized for persistent headache and suspected CNS infection, based on clinical symptoms, elevated intracranial pressure and CSF leukocytosis. She received empirical antiviral and dehydration therapy, which improved symptoms; however, definitive microbiological evaluation and follow-up were not pursued.

During this admission, she presented with poorly controlled diabetes mellitus and developed a triad of neurological symptoms on hospital day 7-11: headache, fever, and generalized tonic-clonic seizures. CSF analysis confirmed *K. pneumoniae* meningitis. A study of 49 patients with *K. pneumoniae* meningitis reported purulent CSF with a mean leukocyte count of 1.42 x 10^9^/L (11) and imaging findings such as meningitis or brain abscess. Our patient’s CSF was jelly-like in appearance, with elevated leukocyte counts. MRI revealed diffuse linear leptomeningeal enhancement—findings consistent with previously reported *K. pneumoniae* meningitis characteristics.

While the diagnosis is clear, the route of bacterial invasion warranted further consideration. *K. pneumoniae* infections are classified as either community-acquired (often involving hypervirulent strains infecting immunocompetent individuals) or healthcare-associated (affecting immunocompromised patients, including those with diabetes) (12). This patient had two major risk factors: a persistent calvarial defect after neurosurgery and uncontrolled diabetes. Her clinical history, imaging, and CSF findings suggest a delayed-onset, healthcare-associated CNS infection, possibly due to chronic colonization following her previous neurosurgical intervention. It is plausible that under sustained hyperglycemia latent colonization progressed to overt infection. This combination of a persistent anatomical defect and an immunosuppressed state likely facilitated the reactivation of a latent focus or *de novo* invasion, leading to the dramatic clinical presentation.

A distinguishing aspect of this case is the longitudinal measurement of 12 inflammatory cytokines in both CSF and peripheral blood. While peripheral blood leukocyte counts normalized rapidly after systemic antibiotic treatment, CSF parameters–including leukocyte count and cytokines such as IL-1β, IL-6, IL-8, IL-2, IL-10, IL-17, IFN-γ, and TNF-α–remained elevated. These markers peaked on day 15; in particular, IL-6, IL-8 and IL-1β rose sharply, consistent with prior reports (13), and declined only after intrathecal antibiotic administration (**Figure 2**). This temporal dissociation between systemic and central inflammation suggests that peripheral markers may not reliably reflect intracranial inflammatory status. Additionally, an interesting temporal pattern of IL-1β was observed. IL-1β tracked closely with CSF leukocyte count (**Figure 2**), whereas other CSF cytokines appeared influenced by the peripheral leukocyte rise between hospital days 20 and 25. Recent evidence suggests that IL-1β-driven macrophage-meningeal fibroblasts axis is a key mediator of inflammatory amplification during pneumococcal central nervous system infection (14). In this patient, CSF IL-1β exhibited temporal dynamics that closely paralleled CSF leukocyte counts. This observation suggests that IL-1β may warrant further investigation as a marker of intracranial inflammatory activity; however, no conclusions regarding its comparative performance relative to other cytokines can be drawn from a single case.

CSF cytokine profiles correlate with disease course and can aid differential diagnosis (15). In a large retrospective cohort of patients evaluated for CNS infection, CSF CRP, IL-6, and IL-1β were significantly elevated, especially in bacterial meningitis; notably, CSF IL-1β (AUC 0.86), IL-6 (AUC 0.93), and TNF-α (AUC 0.82) outperformed leukocyte count (AUC 0.80) for identifying bacterial meningitis (16). Our longitudinal data extend these findings by showing not only the diagnostic elevation of IL-1β but also its potential value in monitoring therapeutic response, as it closely mirrored the resolution of CSF pleocytosis following targeted intrathecal therapy. Moreover, when distinguishing bacterial meningitis from sepsis-associated encephalopathy, CSF biomarkers such as IL-1RA (AUC 0.93), TNF-α (AUC 0.92), CXCL-10 (AUC 0.90), IL-1β (AUC 0.92), IL-6 (AUC 0.94) showed substantially higher concentrations in confirmed or probable meningitis than in sepsis associated encephalopathy (13). Together, these data align with our observation that CSF-focused cytokine dynamics provide information that peripheral parameters may miss.

A 2024 meta-analysis demonstrated that intrathecal therapy improves clinical outcomes, facilitates bacterial clearance, and reduces mortality without increasing adverse event rates when used within recommended dosing limits (9). The patient was treated with polymyxin B and dexamethasone, both intravenously and intrathecally. The treatment was well tolerated, with no adverse events. CSF parameters normalized (**Figure 1**), seizure resolved, and the patient made a full neurological recovery. These findings align with previous studies reporting the benefits of combined intravenous and intrathecal antibiotic therapy in post-neurosurgical Gram-negative meningitis/ventriculitis (17). Intrathecal antimicrobial therapy should generally be regarded as an adjunctive rather than first-line treatment strategy and is most commonly reserved for multidrug-resistant pathogens, healthcare-associated ventriculitis and meningitis, or cases that fail to respond adequately to systemic antimicrobial therapy (18). In the present case, escalation to meropenem antimicrobial administration was undertaken because of persistent CSF pleocytosis, worsening leptomeningeal enhancement on follow-up MRI, and continued isolation of *K. pneumoniae* from CSF cultures despite ongoing antimicrobial treatment. These findings raised concern for ongoing intracranial infection and an inadequate therapeutic response. However, the isolate remained susceptible to multiple conventional antimicrobial agents and did not meet criteria for multidrug resistance. Therefore, the management strategy employed in this case may reasonably be debated. An alternative approach would have been to continue optimized systemic therapy with close clinical, microbiological, and radiographic monitoring before introducing polymyxin B and intrathecal therapy. Such a strategy might have reduced exposure to the potential risks associated with nephrotoxic agents and invasive intrathecal administration. We therefore believe that this case illustrates one possible management approach in a complex clinical scenario rather than establishing a preferred treatment strategy for susceptible *K. pneumoniae* meningitis.

In summary, this case provides comprehensive longitudinal data on CSF cytokine dynamics, neuroimaging progression, and the therapeutic response to intravenous-intrathecal antibiotic therapy. The patient’s recovery underscores the value of early recognition, combined antimicrobial strategies, and cytokine monitoring in severe CNS infections. Furthermore, our serial observations suggest that CSF IL-1β may represent a potentially useful biomarker for monitoring intracranial inflammatory activity. However, this hypothesis requires validation in larger prospective studies.

## Data Availability

The original contributions presented in the study are included in the article/**Supplementary Material**. Further inquiries can be directed to the corresponding author.

## References

[1] Hetta HF Alanazi FE Ali MAS Alatawi AD Aljohani HM Ahmed R . Hypervirulent *Klebsiella* pneumoniae: Insights into virulence, antibiotic resistance, and fight strategies against a superbug. Pharm (Basel). (2025) 18:724. doi: 10.3390/ph18050724 40430542 PMC12115101

[2] Kareem SM Al-Kadmy IMS Kazaal SS Mohammed Ali AN Aziz SN Makharita RR . Detection of gyrA and parC mutations and prevalence of plasmid-mediated quinolone resistance genes in *Klebsiella* pneumoniae. Infect Drug Resist. (2021) 14:555–63. doi: 10.2147/IDR.S275852 33603418 PMC7886241

[3] Al Ismail D Campos-Madueno EI Donà V Endimiani A . Hypervirulent *Klebsiella pneumoniae* (*hvKp*): Overview, epidemiology, and laboratory detection. Pathog Immun. (2024) 10:80–119. doi: 10.20411/pai.v10i1.777 39911145 PMC11792540

[4] Fazili T Sharngoe C Endy T Kiska D Javaid W Polhemus M . *Klebsiella pneumoniae* liver abscess: An emerging disease. Am J Med Sci. (2016) 351:297–304. doi: 10.1016/j.amjms.2015.12.018 26992260

[5] Wyres KL Lam MMC Holt KE . Population genomics of *Klebsiella pneumoniae*. Nat Rev Microbiol. (2020) 18:344–59. doi: 10.1038/s41579-019-0315-1 32055025

[6] GBD 2019 Meningitis Antimicrobial Resistance Collaborators . Global, regional, and national burden of meningitis and its aetiologies, 1990-2019: A systematic analysis for the Global Burden of Disease Study 2019. Lancet Neurol. (2023) 22:685–711. doi: 10.1016/S1474-4422(23)00195-3 37479374 PMC10356620

[7] Pilmis B Péan de Ponfilly G Farfour E Ranc A-G Fihman V Bille E . Epidemiology and clinical characteristics of *Klebsiella* spp. meningitis in France. Infect Dis Now. (2022) 52:82–6. doi: 10.1016/j.idnow.2021.05.006 34091093

[8] Yang X Wang Y Zhao S Huang X Tian B Yu R . Clinical characteristics and prognosis of *Klebsiella pneumoniae* meningitis in adults. Heliyon. (2024) 10:e28010. doi: 10.1016/j.heliyon.2024.e28010 38601552 PMC11004708

[9] Li M-T Wu Q-Q Li J-B Chen J-S . Intrathecal or intraventricular antimicrobial therapy for post-neurosurgical Gram-negative bacillary meningitis or ventriculitis: A systematic review and meta-analysis. Int J Antimicrob Agents. (2024) 63:107033. doi: 10.1016/j.ijantimicag.2023.107033 37967659

[10] Power EA Rechberger JS Gupta S Schwartz JD Daniels DJ Khatua S . Drug delivery across the blood-brain barrier for the treatment of pediatric brain tumors - An update. Adv Drug Delivery Rev. (2022) 185:114303. doi: 10.1016/j.addr.2022.114303 35460714

[11] Chang W-N Huang C-R Lu C-H Chien C-C . Adult *Klebsiella pneumoniae* meningitis in Taiwan: An overview. Acta Neurol Taiwan. (2012) 21:87–96. 22879119

[12] Li X Zhang H Hu H Song X Leng B . Association between type 2 diabetes mellitus and *Klebsiella pneumoniae* colonization: Construction of nomogram model. Am J Transl Res. (2024) 16:7633–44. doi: 10.62347/DZKV8669 39822526 PMC11733326

[13] Groeneveld NS Olie SE Visser DH Snoek L van de Beek D Brouwer MC . Cerebrospinal fluid inflammatory markers to differentiate between neonatal bacterial meningitis and sepsis: A prospective study of diagnostic accuracy. Int J Infect Dis. (2024) 142:106970. doi: 10.1016/j.ijid.2024.02.013 38395221

[14] Beckenbauer P Ercegovac L Christensen G Lagler P Hammerschmidt S Völk S . Macrophage IL-1β turns meningeal fibroblasts into inflammatory amplifiers in pneumococcal infection. Front Immunol. (2026) 17:1808185. doi: 10.3389/fimmu.2026.1808185 42220478 PMC13215843

[15] Kanjilal S Cho TA Piantadosi A . Diagnostic testing in central nervous system infection. Semin Neurol. (2019) 39:297–311. doi: 10.1055/s-0039-1688441 31378866 PMC7117082

[16] Olie SE Staal SL Ter Horst L van Zeggeren IE Man WK Tanck MWT . Diagnostic accuracy of inflammatory markers in adults with suspected central nervous system infections. J Infect. (2024) 88:106117. doi: 10.1016/j.jinf.2024.01.016 38320644 PMC10943182

[17] Chusri S Sakarunchai I Kositpantawong N Panthuwong S Santimaleeworagun W Pattharachayakul S . Outcomes of adjunctive therapy with intrathecal or intraventricular administration of colistin for post-neurosurgical meningitis and ventriculitis due to carbapenem-resistant acinetobacter baumannii. Int J Antimicrob Agents. (2018) 51:646–50. doi: 10.1016/j.ijantimicag.2017.12.002 29241820

[18] Tunkel AR Hasbun R Bhimraj A Byers K Kaplan SL Scheld WM . 2017 Infectious Diseases Society of America’s clinical practice guidelines for healthcare-associated ventriculitis and meningitis. Clin Infect Dis. (2017) 64:e34–65. doi: 10.1093/cid/ciw861 28203777 PMC5848239

