# Supplementary material for: Case Report: A case of postneurosurgery Klebsiella pneumoniae meningitis with longitudinal CSF cytokine profiling and combined intrathecal therapy
Source: Front Immunol. 2026 Oct 15;17:1945662. doi: 10.3389/fimmu.2026.1945662 (PMC13716629; doi:10.3389/fimmu.2026.1945662)
Supplement: Supplementary file 1 [file Supplementaryfile1.pdf]

Supplementary Materials – Zhu, Wu, Xu, Ge, and Dai

Table S1. Antimicrobial susceptibility profile of the cerebrospinal fluid *Klebsiella pneumoniae* isolate

Antibiotic	Method	Result	Interpretation
Cefoperazone/Sulbactam	MIC	≤ 8 μg/ml	S
Ceftazidime	MIC	≤ 0.12 μg/ml	S
Cefuroxime	MIC	4 μg/ml	S
Piperacillin/Tazobactam	MIC	≤ 4 μg/ml	S
Amoxicillin/Clavulanate	MIC	≤ 2 μg/ml	S
Cefepime	MIC	≤ 0.12 μg/ml	S
Ceftriaxone	MIC	≤ 0.25 μg/ml	S
Imipenem	MIC	0.5 μg/ml	S
Ertapenem	MIC	≤ 0.12 μg/ml	S
Amikacin	MIC	≤ 2 μg/ml	S
Levofloxacin	MIC	≤ 0.12 μg/ml	S
Trimethoprim/Sulfamethoxazole	MIC	≤ 20 μg/ml	S
Tigecycline	MIC	≤ 0.5 μg/ml	S
Cefoxitin	MIC	≤ 4 μg/ml	S
ESBL			
Ampicillin	KB	≤ 6 mm	R
Ampicillin/Sulbactam	KB	≤ 15 mm	S
Aztreonam	KB	≤ 26 mm	S
Meropenem	KB	≤ 27 mm	S
Gentamicin	KB	≤ 17 mm	S
Ciprofloxacin	KB	≤ 26 mm	S
Cefazolin	KB	≤ 20 mm	I

Abbreviations: MIC, minimum inhibitory concentration; KB, Kirby-Bauer disk diffusion; S, susceptible; I, intermediate; R, Resistant.

Figure S1. Cranial CT demonstrating a postoperative skull defect.

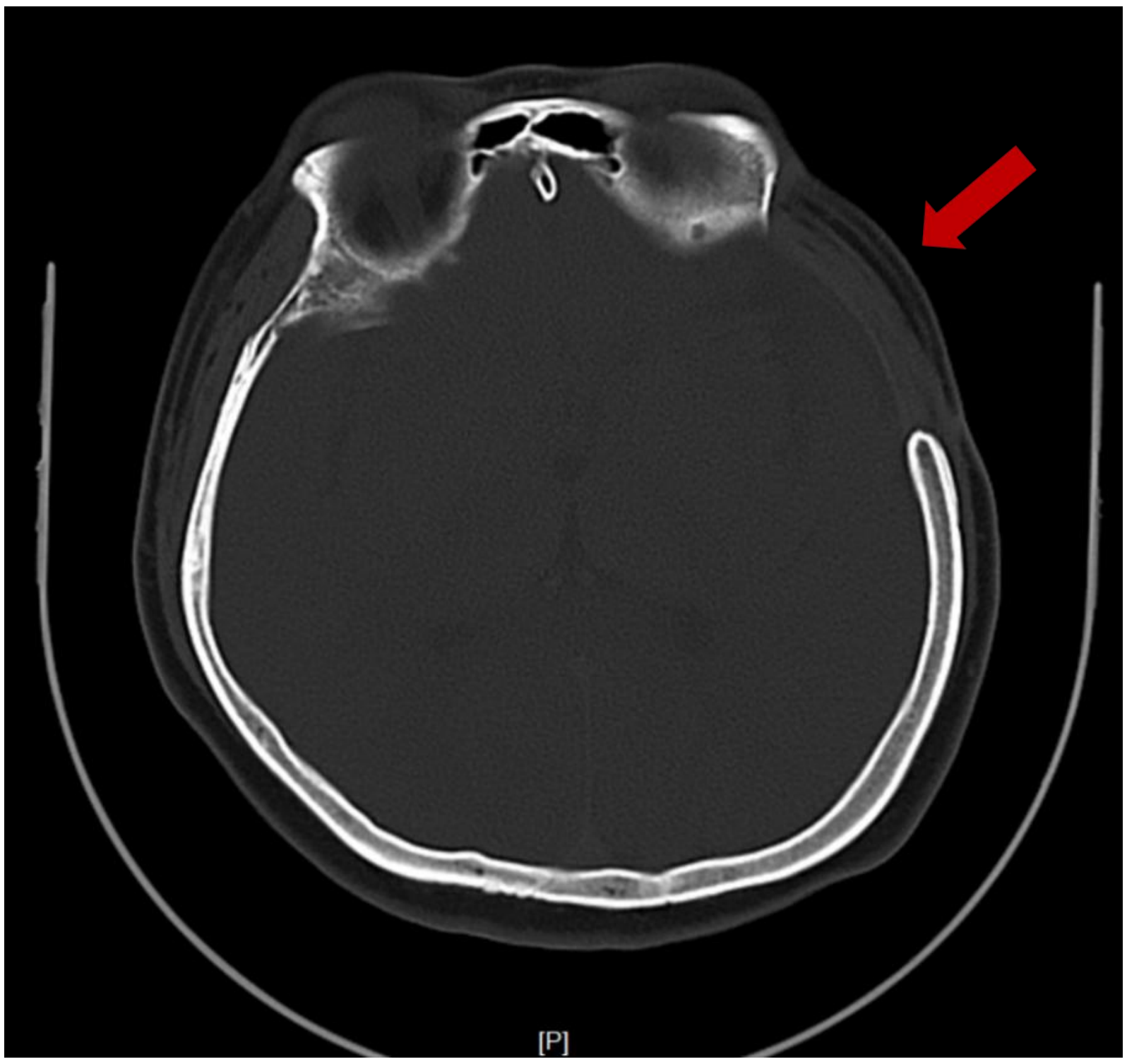

Figure S1. Non-contrast head CT showing a bony defect in the left frontotemporal region (arrow), consistent with previous neurosurgical intervention.

Figure S2. Longitudinal serum creatinine measurements during polymyxin B treatment

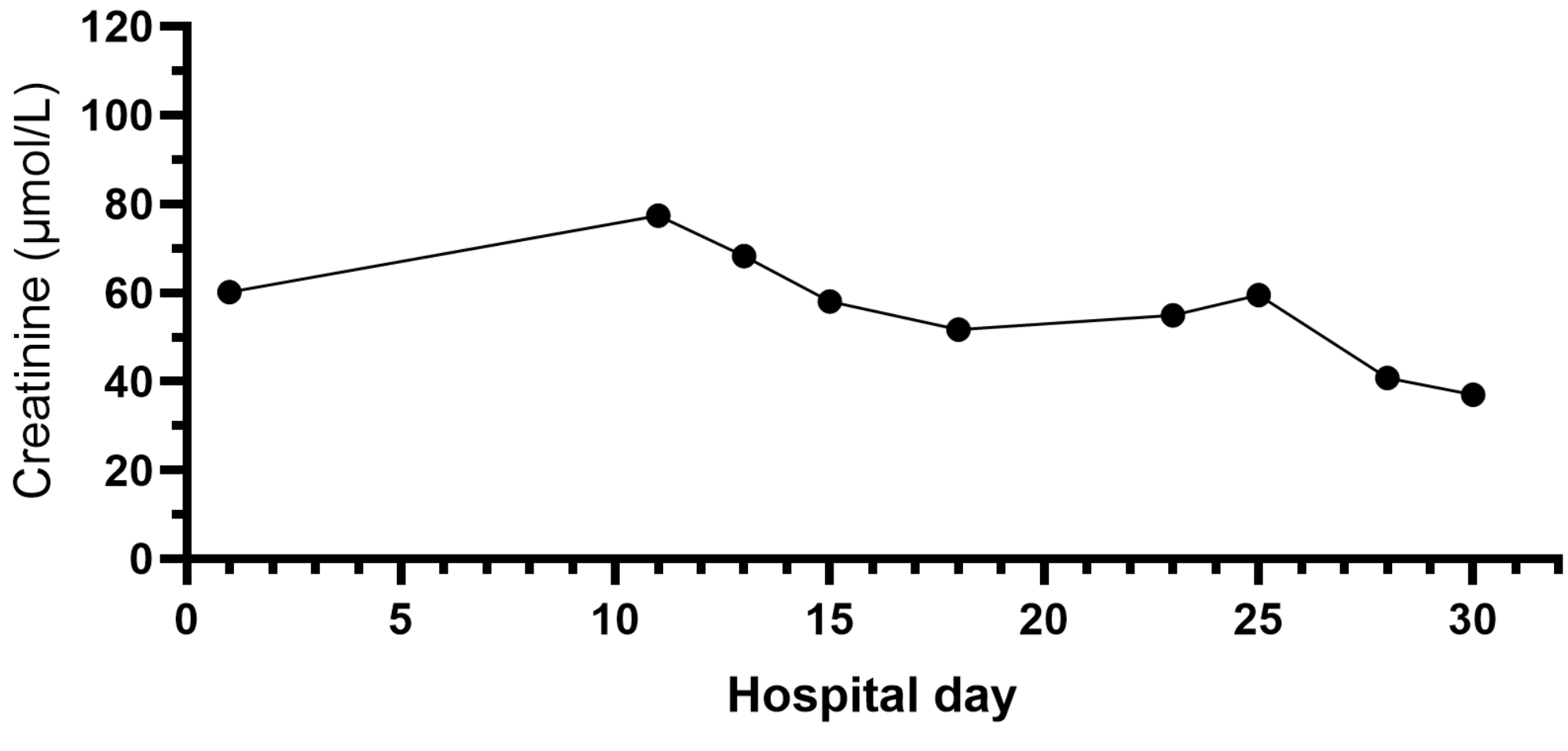

Figure S2. Serial serum creatinine measurements during hospitalization. Intravenous polymyxin B was administered from hospital day 16 to day 24. Serum creatinine (normal range: 41.0 – 73.0 μmol/L) remained stable throughout treatment, and no acute kidney injury or clinically significant renal dysfunction was observed.
